# Detection of *C. trachomatis* in the Serum of the Patients with Urogenital Chlamydiosis

**DOI:** 10.1155/2013/489489

**Published:** 2013-02-13

**Authors:** Naylia A. Zigangirova, Yulia P. Rumyantseva, Elena Y. Morgunova, Lidia N. Kapotina, Lubov V. Didenko, Elena A. Kost, Ekaterina A. Koroleva, Yuriy K. Bashmakov, Ivan M. Petyaev

**Affiliations:** ^1^Department of Medical Microbiology, Gamaleya Institute of Epidemiology and Microbiology, Ministry of Health of the Russian Federation, Gamaleya Street 18, Moscow 123098, Russia; ^2^Lycotec Ltd., St John's Innovation Park, Cowley Road, Cambridge CB4 0WS, UK

## Abstract

Extragenital chlamydial complications may be associated with systemic spread of infection, but haematogenous route for *C. trachomatis* dissemination has not been clearly demonstrated. Here we report that serum specimens obtained from patients with chlamydiosis contain elementary bodies of *C. trachomatis* shown by culture and immunogold electron microscopy. We have found that 31 of the 52 patients had serum precipitates which were infective to McCoy cells. Immunostaining revealed very small inclusions resembling those reported during persistent *C. trachomatis* infection *in vitro*. DNA specimens from 49 (out of 52) patients with chlamydiosis gave positive PCR readings. The viability of the pathogen present in the sera was confirmed by chlamydial RNA detection in the cell monolayer inoculated by the serum precipitates. By using DNA isolation protocol from 1 mL of serum and quantitative TaqMan PCR, it was estimated that bacterial load in patients' sera was 2 × 10^2^–10^3^ GE/mL. These findings for the first time demonstrated that *C. trachomatis* can be disseminated directly by the plasma, independently from blood cell, which may represent a new possible pathway of the chronic infection development. Therefore, new methodological approaches for detection of *C. trachomatis* in the serum of patients with complicated and chronic chlamydiosis could be important in the diagnosis of the infection regardless of its anatomical localization.

## 1. Introduction

*Chlamydia trachomatis* is an obligate gram-negative intracellular bacteria which belongs to genus *Chlamydia*. Three major biovars of *C. trachomatis* are believed to induce different diseases in humans trachoma (serovars A, B, Ba, or C), urethritis, epididymitis, cervicitis, salpingitis and pelvic inflammatory disease (serovars D–K), and lymphogranuloma venereum (LGV, serovars L1, L2, and L3) [1]. Cellular paradigm of chlamydial infection emerges from the fact that epitheliocytes are the main primary target of *C. trachomatis* in the human body [2]. Chemokines released from epithelial cells can recruit neutrophils and a modest number of monocytes which in turn can infiltrate submucosal zone within 24–48 hours after initiation of the infection [3]. Epitheliocytes provide perfect support for full developmental cycle of *C. trachomatis* which concludes in cells lysis and exposure of adjacent epithelial cells to the *de novo* infectious progeny [4]. Horizontal spread of the pathogen within epithelium and further cytokine release from migrated innate immune cells culminate in the appearance of lymphocytes in the site of infection [5]. Viable forms of *C. trachomatis* can be found within monocytes, neutrophils, lymphocytes in epithelial lesions, and as far as in regional lymph nodes [6, 7]. Phagocytosis, pinocytosis, and receptor-mediated uptake are responsible for chlamydial entry into innate immunity cells [8]. According to their ability to spread, *C. trachomatis* serovars are divided into invasive and noninvasive. Serotypes L1, L2, and L3 cause invasive infections, whereas types D, E, F, G, and K restrict their propagation to the site of primary chlamydial insult [9]. Nevertheless, in many cases of *C. trachomatis* infection the pathogen and clinical manifestation of the disease spread far beyond originally infected area. Canicular spread of the pathogen is traditionally believed to be a major way of *C. trachomatis* generalization [2, 3] though this mechanism is not relevant when pathogen is detected in tissues with no anatomical connectivity to urogenital system. Extragenital complications of *C. trachomatis* infection include sexually acquired arthritis (SARA), perihepatitis, and conjunctivitis [10]. Isolation and/or detection of *C. trachomatis* has been reported from synovial exudate [11], ascitic fluid [12, 13], liver biopsy material [14, 15], and respiratory secretion fluids [16]. However, the mechanism of systemic generalisation of chlamydial disease remains yet to be understood. Lymphogenic dissemination is likely to be implicated since *C. trachomatis* propagates in lymphocytes and can be isolated from regional lymph nodes [7, 17]. 

Recently, we showed that *C. pneumoniae* and *C. trachomatis* can be detected in serum specimens obtained from patients [18, 19]. Here we report new data on isolation and identification of *C. trachomatis* viable infectious forms from serum of patients with urogenital chlamydial infection.

## 2. Material and Methods

### 2.1. Patients

The study was conducted in the outpatient clinic NearMedic at Gamaleya Institute of Epidemiology and Microbiology RAMN (Moscow, Russia). The study protocol was approved by the local ethical committee. All patients were informed about the purpose of the study and have given a written consent regarding participation in the study. The major group of the study included 52 patients with newly diagnosed symptomatic chlamydial infection having complaints of abnormal vaginal discharge, genital ulcer, lower abdominal pain (women), or urethral discharge (men). Patients with pelvic inflammatory diseases or epididymitis and other infections (*N. gonorrhoeae*, tuberculosis, HIV, Hepatitis B or C, and syphilis) or patients admitting antibiotic use within 4 weeks before enrollment were excluded from the study. Thirty women (range 19–38 years) and 22 men (range 24–45 years) with diagnosis of urogenital symptomatic *C. trachomatis* infection confirmed by cell culture and PCR were eligible for the study. Control group included 20 age-matched healthy volunteers (9 males range 19–30 years, 11 females 23–40 years) with no history or current evidence of STD and with negative PCR results of swab specimens. Groups of 36 patients with chronic pelvic inflammatory diseases (range 28–41 years), 33 patients with Reiter's disease (range 31–46 years), and 20 control subjects (range 26–50 years) were examined for chlamydial DNA presence in serum and urogenital swabs. The study neither interfered with diagnostic and therapeutic options nor had impact on treatment options chosen by physicians for each consented individual.

### 2.2. Specimen Handling

#### 2.2.1. Serum

Blood was collected in the morning hours (8–11 AM) under sterile conditions from fasted patients by venipuncture. After clotting the blood for 1 hour at room temperature, the tubes were centrifuged at 1600 g for 15 min at 4°C. Serum was separated immediately and 3 mL was subjected to the centrifugation on Beckman centrifuge AN (Beckman Coulter, Inc., USA) at 16000 g for 60 min at 4°C. Obtained sediments were gently resuspended with micropipette in 0.5 mL of SPG and stored at –80°C. Vials were opened only in the laminar air flow safety cabinet for culture test and PCR analysis after confirmation of diagnosis of chlamydial infection.

#### 2.2.2. Urogenital Swabs

Urethral and cervical swabs for bacteriological culture and DNA amplification were collected by qualified professionals using commercial swabs (Aptaca, China). Swabs were placed in the tubes containing RPMI-1640 medium with 5% FCS, amphotericin B (5 *μ*g/mL), and gentamycin (4 *μ*g/mL) and were frozen at −80°C until cultivation.

### 2.3. Reagents and Bacteria

All reagents were purchased from Sigma-Aldrich unless specifically mentioned otherwise. *C. trachomatis* strain L2/Bu434 kindly provided by Dr. P. Saikku (University of Oulu, Finland) was used as reference culture. The pathogen was propagated in *Mycoplasma*-free McCoy cells grown in RPMI-1640 medium supplemented with 2 mM L-glutamine (Invitrogen), 5% fetal bovine serum, 50 *μ*g/mL gentamycin sulfate, and 1 mg cycloheximide/mL. Infectious elementary bodies were isolated from McCoy cells by sonication, washed in phosphate-buffered saline, purified by Renografin gradient centrifugation, and kept frozen at −80°C in SPG buffer (pH 7.2; 250 mM sucrose, 10 mM sodium phosphate, 5 mM L-glutamic acid).

### 2.4. Cell Culture Test

#### 2.4.1. Urogenital Swabs

Thawed swabs were vortexed vigorously and centrifuged at 300 g for 3 min. Resulting supernatant was added to 24 well plates with subconfluent McCoy cells. The plates were centrifuged at 1600 g for 1 hour at 30°C and incubated for 2 h at 37°C in 5% CO_2_. The medium was removed and replaced with fresh RPMI-1640 and plates were cultivated for 48 hours at 37°C in 5% CO_2_. Infected cells were visualized by direct immunofluorescence with FITC-labeled antibodies against *C. trachomatis* MOMP (NearMedic Plus, Russia). The identity of propagating bacteria was confirmed by TaqMan—PCR conducted with DNA extracted from infected McCoy cells monolayers.

#### 2.4.2. Serum

Tubes containing 0.5 mL of frozen serum sediments in SPG were thawed on ice, then 2.5 mL of RPMI-1640 supplemented with FCS, amphotericin B and, gentamycin was added to each tube. Resulting suspension was transferred to 3 wells of subconfluent monolayers of McCoy cells grown in 24 well plates. Plates were incubated and processed as described above for urogenital swabs. Remaining serum was used for DNA extraction and further PCR analysis.

*C. trachomatis*-specific IgG antibodies were evaluated by using *Chlamydia trachomatis*-IgG-ELISA plus medac commercial kit (Medac, Hamburg, Germany).

### 2.5. Immunofluorescence Staining

Infected McCoy cell monolayers were grown on coverslips in 24 well plates. After fixating with methanol and permeabilization with Triton X-100, cells were stained by direct immunofluorescence with FITC—conjugated monoclonal antibody against *C. trachomatis* MOMP (NearMedic Plus, Russia). Inclusion-containing cells were examined and photographed under Nikon Eclipse 50i fluorescent microscope at 1350x magnification.

### 2.6. Electron Microscopy

Thawed serum samples (10 mL) were spun at 16000 g for 60 min. Resulting pellets were analyzed by TaqMan PCR for *C. trachomatis* 16S rRNA. Positive specimens were fixed for 4 hours in phosphate buffer (pH 7.8) containing 5% glutaraldehyde, postfixed in 1% osmium tetroxide for 1 hour, dehydrated in ethanol, and embedded in LR White resin (EMS, USA). Stained ultrathin sections (200–300 Å) were evaluated by electron microscopy using JEM-100B microscope (Japan Electron Optics Laboratory Co., Tokyo, Japan). Purified EBs of *C. trachomatis* reference strain were used as positive control for electron microscopy studies. PCR-negative sediments of serum obtained from healthy volunteers served as negative control.

### 2.7. DNA Isolation

Extraction of total nucleic acids was conducted with IVD-labeled automated system NucliSENS easyMAG (BioMérieux Inc., The Netherlands). DNA was isolated from urogenital swaps, serum specimens, and McCoy cells' infected serum aliquots. Up to 24 samples were analyzed in one BioMérieux automated system run. All specimens with the adjusted volume of 1.0 mL were treated with 1 mL of BioMérieux lysis buffer. Loadings of samples, reagents, and disposables were the only manual steps during DNA extraction procedure using NucliSENS easyMAG platform. DNA was eluted from the cartridges with 50 *μ*L of BioMérieux elution buffer.

### 2.8. Quantitative TaqMan PCR

For quantification purpose, real-time PCR for 16S rRNA and cryptic plasmid of *C. trachomatis* was conducted. PCR primers and TaqMan probe for 16S rRNA of *C. trachomatis* (GenBank accession number AM884176) and cryptic plasmid of *C. trachomatis* (GenBank accession number X06707.3) were designed using Primer Express Software (Applied Biosystems, Foster City, CA, USA). Designed primers and TaqMan probe were as follows: for 16S rRNA of *C. trachomatis* forward primer, 5′-GGC GTA TTT GGG CAT CCG AGT AAC G-3′; reverse primer, 5′-TCA AAT CCA GCG GGT ATT AAC CGC CT-3′; and TaqMan probe R6G-TGG CGG CCA ATC TCT CAA TCC GCC TAG A-BHQ2, and for cryptic plasmid of *C. trachomatis* forward primer, 5′-GGG ATT CCT GTA ACA ACA AGT CAG G-3′; reverse primer, 5′-CCT CTT CCC CAG AAC AAT AAG AAC AC-3′; and TaqMan probe ROX-CTC CCA GAG TAC TTC GTG CAA GCG CTT TGA-BHQ2. An additional BLAST search analysis was conducted to unsure specificity of the primers and probe. Real-time PCR was performed with the iCycler IQ system (Bio-Rad, USA). All samples were analyzed in triplicates.

Bacterial load in serum specimens and urogenital swabs is shown below in genome equivalents of per mL of serum or in genome equivalents of the pathogens referred to 10^5^ copies of eukaryotic *β*-actin (swabs). Calibrator standards were prepared using amplified fragments of 16S rRNA and pLGV440 of *C. trachomatis*, or eukaryotic *β*-actin and cloning them into the pGEM-T plasmid vector (pVU56) using the TA cloning kit (Invitrogen, San Diego, CA, USA) similarly to Broccolo's protocol patients [20].

### 2.9. RNA Isolation

RNA was isolated from infected McCoy cells using Trizol protocol (Invitrogen, USA). RNA preparations were treated with RNase-free DNase RQ1 (Promega Corporation, USA) and resuspended in DEPC-treated water. RNA samples were confirmed to be DNA free by performing a PCR for selected *C. trachomatis* genes. The concentration of isolated RNA in samples was established by spectrophotometry.

### 2.10. Reverse Transcription and PCR

cDNA was synthesized using “Reverse Transcription System” (Promega Corporation, USA) with random primer. PCR has been performed using sets of primers for 4 *C. trachomatis* genes as follows:

 groEL-forward 5′-TCTGCGAACGAAGGATATGA-3′ and reverse 5′-ATAGTCCATTCCTGCGCCAGG-3′;
 omp1-forward 5′-CGTTCGTTGCAGACTTACCA-3′ and reverse 5′-GTTCCTCGCATACCGAATGT-3′;
 pmpD-forward 5′-GTTAGACCAAATTCGAGATC-3′ and reverse 5′-AAGATTCTCCGTCACGAGGA-3′;
 omcB-forward 5′-CTGCAACAGTATGCGCTTGT-3′ and reverse 5′-CACGCTGTCCAGAAGAATGA-3′.

PCR products were separated on 1.5% agarose gels using ethidium bromide staining. *C. trachomatis* L2/434/Bu was used as positive control.

## 3. Results and Discussion

**Figure 1:**
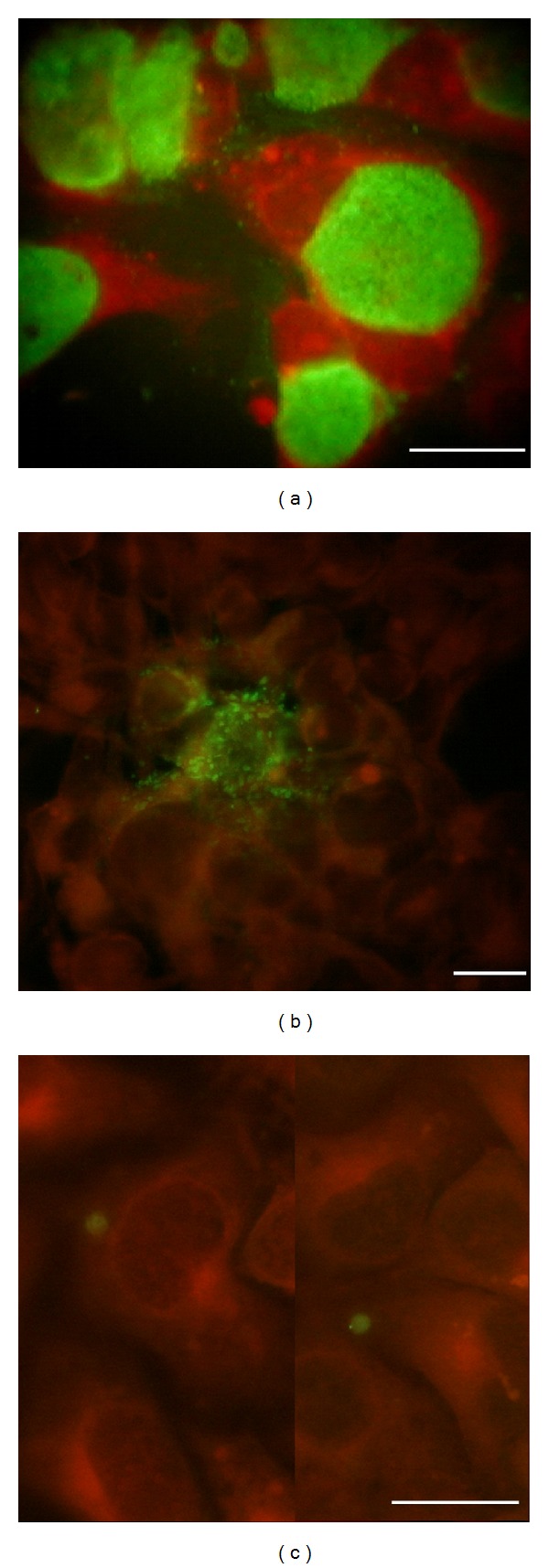
Chlamydial inclusion bodies in McCoy cells infected with reference strain (a), urogenital (b), and serum (c) isolates of *C. trachomatis*. 48 hpi. Bar marker 5 mkm.

As can be seen from Figure 1(a), 48-hour inclusions of *C. trachomatis* reference strain had irregular shape with granular or punctuate IF signal. Some inclusions were stained heterogeneously and contained distinct “empty” pockets that appeared to be deficient in immune-reactive substance. There were many small extracellular particles spread around individual cells which may be indicative of chlamydial inclusion burst.

Inoculation of urogenital swabs obtained from the patients with confirmed chlamydiosis into McCoy cell monolayers led to appearance of inclusions with very variable morphology. Besides typical inclusions, there were many inclusions with irregular borders and reduced intensity of IF signal. In some cells, inclusions retained granular and punctuate IF prototype even at 48 h of postinfection period (Figure 1(b)). Despite variable inclusion morphology, all urogenital swabs from 52 patients with chlamydiosis were culture positive. No specific IF staining has been seen with swab specimens collected in control group except some background signals originated apparently by contaminating blood constituents.

Next, we determined if serum sediments obtained from the same patients with confirmed chlamydiosis might contain infectious forms of *C. trachomatis*. We have found that thirty-one of the 52 patients had serum sediments infective to McCoy cells under conditions used. Immunostaining seen in 48 h of postinoculation had remarkably distinctive pattern (Figure 1(b)). Chlamydial inclusions were fewer in number, smaller, rounder, and more uniformly stained if compared to those seen in McCoy cells infected with reference strain or urogenital swabs (Figure 1). IF signal was commonly weaker and was best seen at 72 h of postinoculation. Most of the inclusions were intact with no extracellular particles extruded suggesting that infectious cycle was not lytic. Overall appearance of IF staining resembled that reported during persistent *C. trachomatis* infection *in vitro* [21].

Ten primary serum isolates sustained at least three serial passages in cell culture. In 9 cases, inclusion morphology remained principally unchanged indicating that these serum isolates had a stable phenotype. Only one serum isolate reversed morphological appearance by showing multiple punctuate pattern of inclusions with extracellular particles on the third passage.

The identity of infectants propagating in McCoy cells was also examined with nucleic acid amplification protocol. Genetic markers of *C. trachomatis* were detected in all 52 DNA specimens from McCoy cells infected with urogenital swabs of patients with chlamydiosis. However, when matching serum sediments were inoculated to McCoy cells, only 49 DNA specimens (out of 52) gave positive PCR readings. Therefore, nucleic acid amplification protocol showed higher detection rate (94.2%) of *C. trachomatis* in McCoy cell infected with serum sediments when compared to IF culture test (59.6%). Simultaneous detection of the pathogen in serum sediments by two methods took place in 30 patients only. Noteworthy that 1 serum specimen inoculated to McCoy cells gave positive IF signal whilst it was negative in PCR. On the other hand, 19 serum sediments which were negative by culture test had PCR-detectable chlamydial markers in the cell monolayers. Positive PCR findings were reproducible for any particular DNA specimen. No positive results were obtained in the control group. Amplicons from two PCR positive samples were sequenced. Full homology was established for chosen region of 16S rRNA gene among two isolates and two reference strains of *C. trachomatis* (L2/434/Bu and A/HAR-13).

**Figure 2:**
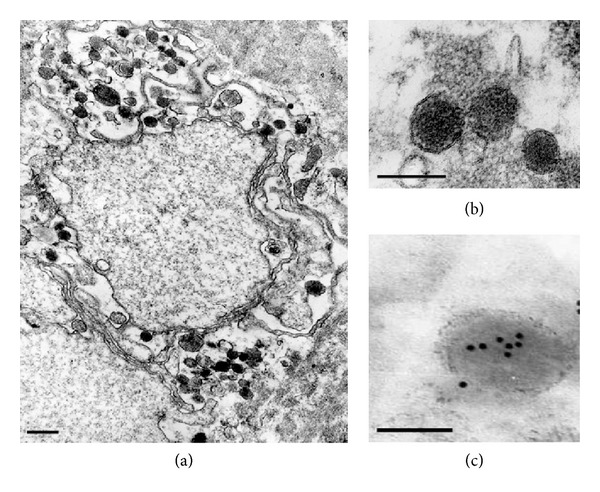
Electron-microscopic images of *C. trachomatis* elementary bodies obtained from serum centrifugates ((a) and (b)). Bar marker 0.5 mkm. Immunogold labeling of *C. trachomatis* elementary bodies after preincubation with monoclonal antibody against chlamydial MOMP (c). Bar marker 0.1 mkm.

The identity of serum isolates from urogenital patients was further evaluated with immunogold electron microscopy using *C. trachomatis* specific antibody. Duplicates of two serum specimens tested positively in culture test and PCR were chosen randomly for retrospective microscopic analysis. Both serum specimens from the patients with urogenital *C. trachomatis* infection contained typical elementary bodies with attached immunogold particles disclosing the identity of the pathogen (Figure 2).

Next, we decided to assess the viability of *C. trachomatis* primary serum isolates by direct comparison of bacterial DNA in inoculums and infected host cells after 48 hours of cultivation of the same volume. Five serum specimens yielding positive identification in culture test and PCR were selected for this purpose. As can be seen from Table 1, there is a significant difference in values of bacterial load measured in inoculums and McCoy cells in 48 hours of postinfection period suggesting effective pathogen propagation in the host cells.

**Figure 3:**
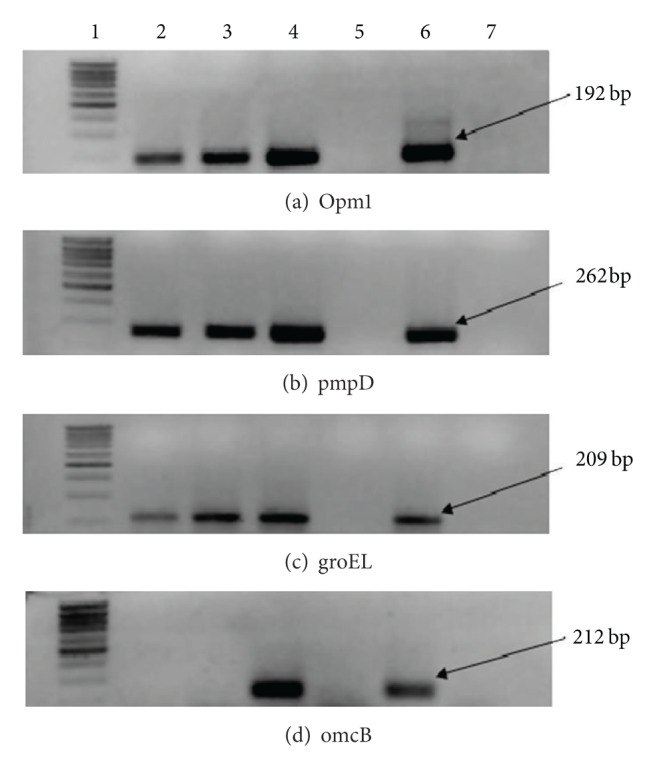
*C. trachomatis*-specific amplicons recovered in RT-PCR reactions with RNA isolated from serum isolates of the patients with urogenital chlamydiosis. RNA was isolated from infected McCoy and RT-PCR reaction performed as described in Section 2. A percentage of 1.2% agarose gel contains the following lanes: 1: size marker; 2 and 3: amplification products obtained in PCR with RNA isolated from serum of patient no. 1 and patient no. 2; 4: amplification product using RNA from reference strain L2/434/Bu; 5: RNA extraction control; 6: positive control; 7: negative control.

In order to reconfirm the viability of pathogen present in serum specimens, we also have been trying to identify chlamydial RNA transcripts in cell monolayer inoculated with serum sediments of the patients with urogenital infections and cultivated for 48 hours. Quantification attempts of *C. trachomatis* RNAs in these specimens were unsuccessful due to extremely high *C*_*t*_ values in TaqMan PCR which is attributable to low bacterial load. We examined the presence of 4 chlamydial transcripts by conventional PCR. Genetic markers of the early (*groEL*), middle (*omp1* and *pmpD*), and late (*omcB*) developmental cycle were chosen. RNA transcripts for *groEL*, *omp1*, and *pmpD* were detectable in total RNA obtained from McCoy cells after inoculation with serum sediments (Figure 3). However, RNA for *omcB* was undetectable in cultured serum isolates suggesting prolonged developmental cycle of serum-derived pathogen.

Next, we determined the possibility of direct detection of chlamydial DNA in patients serum. Using DNA isolation protocol from 1 mL of serum and quantitative TaqMan PCR, it has been shown that bacterial load in patients sera was 2 × 10^2^–10^3^ GE/mL. 

**Figure 4:**
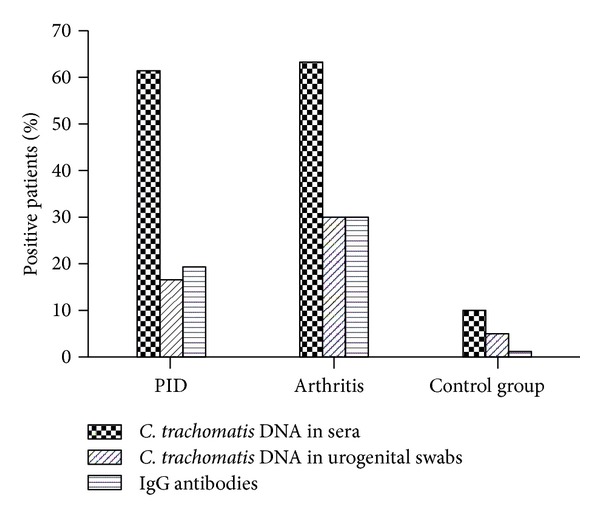
Patients with PID and Reiter's disease and control subjects were examined for presence of *C. trachomatis* DNA in serum and urogenital swab, and *C. trachomatis*-specific IgG antibodies as described in Section 2.

We used the developed protocol for DNA detection in serum to examine the prevalence of *C. trachomatis* infection in patients with chronic urogenital and extragenital complications. Thirty-six patients with chronic pelvic inflammatory diseases, 33 patients with Reiter's disease, and 20 control subjects were examined by simultaneous detection of *C. trachomatis* DNA in urogenital swab and sera. In patients with chronic pelvic inflammatory diseases and a history of chlamydial infection, *C. trachomatis* DNA was detected in the serum of 61% of cases, while in the swabs pathogen was detected in only 17%. In patients with Reiter's disease, *C. trachomatis* DNA was detected in the serum of 64% and 30% in the swabs. In control group chlamydial DNA was found in 7% serum and in 3% swabs (Figure 4).

*C. trachomatis* is an obligate intracellular human pathogen accounting for most of the cases of STDs and preventable blindness in the world. Mucosal surfaces of urogenital system and conjunctivae are the primary target of the pathogen in the human body [2]. However, there is a growing body of evidence that chlamydial infection can spread far beyond mucosal membranes affecting some abdominal organs and joints [10]. Pathophysiological mechanisms of *C. trachomatis* infection generalisation remain obscure [3, 10]. In this paper, we report that viable elementary bodies of *C. trachomatis* can be detected in serum of the patients with newly diagnosed cases of urogenital chlamydial disease.

Firstly, immunogold electron microscopy and cell culture analysis revealed that 14000 g pellet fraction of serum specimens obtained from the patients contains elementary bodies of *C. trachomatis*.

Secondly, 59.6% of serum specimens were tested positively in IF culture test suggesting viability of bacteria. Inclusions originated in McCoy cells by inoculation of serum specimens were remarkably atypical. Inclusions were fewer in number, smaller, round-shaped and had reduced intensity of IF staining when compared side by side with reference strain of *C. trachomatis* propagating in host cells. However, culture test alone seems to underestimate the bacteraemia rate in urogenital patients. When PCR for 16S rRNA and cryptic plasmid of *C. trachomatis* was applied for culture test, estimated detection rate of the pathogen in serum was 94.2%. This finding itself reveals presence of viable pathogen in serum since extrinsic chlamydial DNA is unstable. Coincident detection of *C. trachomatis* in serum specimens by both methods (culturing with further PCR) took place in 57.7% of the patients with urogenital chlamydiosis. No specific IF staining or valid PCR readings with *C*_*t*_ < 40 were seen in serum specimens from control patients. Amplicon sequencing confirmed the identity of the pathogen propagating in McCoy cells after inoculation of serum sediments.

Thirdly, *C. trachomatis* serum isolates display reasonable replicative capacities. There was net increase of bacterial copy number in infected McCoy cells over incubation period when postincubation bacterial load values were compared to PCR readings in the inoculums. It is clear that *de novo* formed chlamydial particles rather than the carryover from the inoculum are responsible for such an increase. Convincing evidence comes from RNA analysis. Genetic markers of chlamydial developmental cycle (*groEL*, *omp1*, and *pmpD*) were expressed in the cultured cells after inoculation of serum specimens.

Direct analytical quantification of *C. trachomatis* genetic markers in serum specimens by nucleic acid amplification reactions needs to be addressed in further studies. Although there are detectable chlamydial target sequences in DNA specimens from plasma of the patients with urogenital chlamydiosis, high *C*_*t*_ readings undermine analytical value of the assay. In search for another target sequence, multiplexed format with two calibrators might be useful.

Therefore our data above suggests that serum specimens of the patients with urogenital chlamydiosis may contain viable forms of *C. trachomatis*. Although diagnostic evaluation of this finding needs to be assessed in further larger trials, our data already indicates a high level of prevalence of the detection of this bacteria in patients with chlamydial diseases. Moreover, we have recently published that another chlamydial species, *C. pneumonia*, can be detected in serum specimens of the patients with acute coronary syndrome [18]. It seems that the detectability, that is, presence of chlamydial pathogens in the serum, and their circulation in the blood, is quite a common feature attributable to different chlamydial diseases, indicating similarity in mechanisms of dissemination of these two pathogens. Epitheliotropism as well as ability to infect mononuclear cells and lymphocytes is another common feature shared by both pathogens in terms of pathogenesis of chlamydial disease.

Our study did not reveal precisely whether pathogen present in serum has originated from mononuclear cells or plasma. However, it becomes clear from our work that even early stages of chlamydial urogenital disease are accompanied by presence of culturally retrievable *C. trachomatis* in blood predisposing to systemic dissemination of the pathogen in the human body.

## 4. Conclusions 

It was demonstrated for the first time that *C. trachomatis* can, independently from blood cells, be disseminated by circulation which may lead to the development of chronic infection. Taken together, our data suggest that serum specimens of the patients with urogenital chlamydiosis contain viable forms, elementary bodies of *C. trachomatis*, capable of initiation of new infectious cycle in the host cells. The new methodological approach of detection of *C. trachomatis* in the serum of patients with complicated chlamydiosis could be important in the direct diagnosis of the infection regardless of its localization and for evaluation of the effectiveness of treatment based on quantitative estimation of bacterial load.

## Figures and Tables

**Table 1:** Comparison of amounts of *C. trachomatis* genome equivalents (GEs), detected by direct isolation from the serum and after cultivation in cell culture using PCR.

Patient	*C. trachomatis* GEs in 1 mL of serum	*C. trachomatis* GEs isolated from cells after cultivation of 1 mL serum inoculum for 48 h
2	2.54∗10^2^	1.36∗10^4^
6	8.21∗10^2^	2.54∗10^4^
9	4.21∗10^2^	4.41∗10^4^
15	2.78∗10^2^	3.27∗10^4^
19	3.05∗10^2^	9.21∗10^4^
